# Whole genome sequencing to identify predictive markers for the risk of drug-induced interstitial lung disease

**DOI:** 10.1371/journal.pone.0223371

**Published:** 2019-10-04

**Authors:** Chihiro Udagawa, Hidehito Horinouchi, Kouya Shiraishi, Takashi Kohno, Takuji Okusaka, Hideki Ueno, Kenji Tamura, Yuichiro Ohe, Hitoshi Zembutsu

**Affiliations:** 1 Division of Genetics, National Cancer Center Research Institute, Tokyo, Japan; 2 Department of Thoracic Oncology, National Cancer Center Hospital, Tokyo, Japan; 3 Division of Genome Biology, National Cancer Center Research Institute, Tokyo, Japan; 4 Department of Hepatobiliary and Pancreatic Oncology, National Cancer Center Hospital, Tokyo, Japan; 5 Department of Breast and Medical Oncology, National Cancer Center Hospital, Tokyo, Japan; 6 Project for Development of Liquid Biopsy Diagnosis, Japanese Foundation for Cancer Research, Research Institute, Tokyo, Japan; Children's Cancer Hospital-57357-Egypt, EGYPT

## Abstract

Drug-induced interstitial lung disease (DIILD) is a serious side effect of chemotherapy in cancer patients with an extremely high mortality rate. In this study, to identify genetic variants with greater risk of DIILD, we carried out whole genome sequencing (WGS) of germline DNA samples from 26 patients who developed DIILD, and conducted a case-control association study between these 26 cases and general Japanese population controls registered in the integrative Japanese Genome Variation Database (iJGVD) as a screening study. The associations of 42 single nucleotide variants (SNVs) showing *P* < 0.0001 were further validated using an independent cohort of 18 DIILD cases as a replication study. A further combined analysis of the screening and replication studies showed a possible association of two SNVs, rs35198919 in intron 1 of the chromosome 22 open reading frame 34 (*C22orf34*) and rs12625311 in intron 1 of the teashirt zinc finger homeobox 2 (*TSHZ2*), with DIILD (*P*_*combined*_ = 1.87 × 10^−5^ and 5.16 × 10^−5^, respectively). Furthermore, in a subgroup analysis of epidermal growth factor receptor (EGFR)–tyrosine kinase inhibitor (TKI)-induced interstitial lung disease (ILD), we observed seven candidate SNVs that were possibly associated with ILD (*P* < 0.00001). This is the first study to identify genetic markers for the risk of DIILD using WGS. Collectively, our novel findings indicate that these SNVs may be applicable for predicting the risk of DIILD in patients receiving chemotherapy.

## Introduction

Drug-induced interstitial lung disease (DIILD) is a serious side effect of a wide range of drugs, including anticancer [1–3], antirheumatic [4–6], and cardiology drugs [7–9]. While the incidence rate of DIILD is approximately 0.1%–10% for anticancer drugs, DIILD is potentially life-threatening and represents a serious clinical problem [10]. Therefore, tools for predicting the risk of DIILD are important in order to avoid life-threatening drug toxicity. Although the precise mechanism underlying DIILD is not well understood, cumulative evidence suggests that direct cytotoxic effects related to the parent drug and/or its metabolites, along with certain immune factors, may be involved [11–13]. Serum levels of Krebs von der Lungen-6 (KL-6), surfactant protein (SP) -A and SP-D have commonly been used as biomarkers for ILD in clinical settings [14–16]. KL-6 is a high molecular weight glycoprotein secreted by proliferating or damaged type II alveolar pneumocytes and bronchial epithelial cells and has high diagnostic sensitivity (93.9%) and specificity (96.3%) for ILD [17]. Clinical risk factors for developing DIILD vary according to the disease or drug involved; however, certain risk factors such as increased age [18–20], preexisting ILD or idiopathic pulmonary fibrosis (IPF) [21, 22], smoking [19, 22], and poor performance status [19, 20] are associated with the development of the toxicity. Although human leukocyte antigen (HLA) alleles have been reported to be associated with methotrexate-induced ILD and gemcitabine plus erlotinib-induced ILD [23, 24], a clinically useful biomarker for predicting the risk of DIILD before treatment has yet to be developed.

In this study, we carried out whole genome sequencing (WGS) on genomic DNA from 26 patients who developed DIILD and identified two genomic regions, the chromosome 22 open reading frame 34 (*C22orf34*) and the tea shirt zinc finger homeobox 2 (*TSHZ2*) gene on chromosome 20, which may be associated with DIILD. Our results may provide insight into the underlying mechanism of DIILD and aid in the development of a diagnostic system for predicting the risk of this life-threatening adverse event.

## Materials and methods

### Patients

To identify genetic susceptibility loci for DIILD, we used 26 DIILD patients treated with epidermal growth factor receptor (EGFR)–tyrosine kinase inhibitor (TKI), nivolumab, gemcitabine, and trastuzumab for the screening study. Of the 26 patients, 12 who had EGFR-TKI-induced ILD between June 2002 and July 2008 at the National Cancer Center Hospital, and 1 patient who was registered in the BioBank Japan (http://biobankjp.org/) and satisfied the following criteria; a. EGFR-TKI was used, b. patient with DIILD during the therapy, were used for the screening study. In addition, from the registered samples in the National Cancer Center (NCC) biobank, 13 patients who were treated with nivolumab, gemcitabine, or trastuzumab, and were confirmed to have DIILD in clinical data as of May 2016, were used for the screening study (Table 1). In a replication study, 18 patients who were treated with nivolumab or trastuzumab and were newly confirmed to have DIILD in updated clinical data as of November 2018, were used from the registered samples in the NCC biobank (Table 1). DIILD was diagnosed by clinical course, objective findings, and independent computed tomography (CT) findings by radiologists or respiratory oncologists.

**Table 1:** Demographics of the 44 patients with drug-induced interstitial lung disease (DIILD).

Patient	Tumor	Drug	Smoking history/status	Preexisting ILD	Treatment for ILD	Study set
1	Lung	Gefitinib	Yes / current	No	DX + Oxygen	Screening
2	Lung	Gefitinib	Yes / former	Yes	PSL + Oxygen + CAZ	Screening
3	Lung	Erlotinib	No	Yes	MP + Oxygen + CFPM	Screening
4	Lung	Gefitinib	Yes / former	No	PSL + Oxygen + MEPM	Screening
5	Lung	Gefitinib	No	No	MP + Oxygen + MEPM + Warfarin	Screening
6	Lung	Gefitinib	Yes / former	Yes	PSL + Oxygen + CAZ	Screening
7	Lung	Erlotinib	Yes / former	Yes	DX	Screening
8	Lung	Gefitinib	Yes / former	No	PSL + Oxygen + BIPM	Screening
9	Lung	Gefitinib	No	No	MP + Oxygen + CAZ	Screening
10	Lung	Gefitinib	Yes / former	No	PSL + Oxygen + ST	Screening
11	Lung	Gefitinib	Yes / former	No	MP + Oxygen + PARM/BP	Screening
12	Lung	Gefitinib	Yes / former	Yes	PSL + Oxygen	Screening
13	Lung	Nivolumab	Yes	No	PSL + Oxygen	Screening
14	Lung	Nivolumab	Yes / former	No	MP + Oxygen + Carbocisteine + AZM + CAM	Screening
15	Lung	Nivolumab	Yes / former	No	Oxygen + Warfarin	Screening
16	Lung	Gefitinib	Unknown	Unknown	Unknown	Screening
17	Pancreas	Gemcitabine	Unknown	No	None	Screening
18	Pancreas	Gemcitabine	No	No	None	Screening
19	Pancreas	Gemcitabine	Yes	No	PSL + Ambroxol + ST + Cilostazol	Screening
20	Pancreas	Gemcitabine^a^	No	No	None	Screening
21	Pancreas	Gemcitabine + nab-PTX	No	No	PSL + CEZ	Screening
22	Pancreas	Gemcitabine + nab-PTX	No	No	PSL + Oxygen + TAZ/PIPC	Screening
23	Breast	Trastuzumab + paclitaxel	No	No	PSL + Oxygen + SBTPC + Warfarin	Screening
24	Breast	Trastuzumab^b^	No	No	ST	Screening
25	Breast	Trastuzumab + paclitaxel	No	No	None	Screening
26	Gastric	Trastuzumab + paclitaxel	Yes / former	No	PSL + Oxygen + ICS/LABA + ST	Screening
27	Lung	Nivolumab	Yes / unknown	No	PSL + CPFX	Replication
28	Lung	Nivolumab	Yes / unknown	No	PSL + ST	Replication
29	Lung	Nivolumab	Yes / former	No	PSL + ST	Replication
30	Lung	Nivolumab	Yes / unknown	No	PSL + Oxygen + ST	Replication
31	Lung	Nivolumab	Yes / former	No	PSL + ST	Replication
32	Lung	Nivolumab	Unknown	No	Eprazinone + AZM	Replication
33	Lung	Nivolumab^c^	Yes / unknown	No	MP + Oxygen + TAZ/PIPC + ST	Replication
34	Melanoma	Nivolumab	No	Yes	PSL + ST	Replication
35	Melanoma	Nivolumab	Yes / unknown	No	PSL	Replication
36	Melanoma	Nivolumab	No	No	PSL + Oxygen + ST	Replication
37	Melanoma	Nivolumab	Unknown	No	BMZ + Oxygen + ST	Replication
38	Melanoma	Nivolumab	No	Yes	PSL + ST	Replication
39	Breast	Trastuzumab + paclitaxel	No	No	PSL	Replication
40	Breast	Trastuzumab + paclitaxel	No	No	PSL	Replication
41	Breast	Trastuzumab + paclitaxel	No	No	PSL + CPFX	Replication
42	Breast	Trastuzumab + paclitaxel	No	No	PSL + Budesonide + ST	Replication
43	Gastric	Trastuzumab^d^	No	No	PSL + Oxygen + MEPM	Replication
44	Gastric	Trastuzumab emtansine	No	No	MP + Oxygen + TAZ/PIPC	Replication

DX, Dexamethasone; PSL, Prednisolone; CAZ, Ceftazidime; MP, Methylprednisolone; CFPM, Cefepime; MEPM, Meropenem; BIPM, Biapenem; ST, Sulfamethoxazole trimethoprim; PARM/BP, Panipenem/betamipron; AZM, Azithromycin; CAM, Clarithromycin; CEZ, Cefazolin; TAZ/PIPC, Tazobactam/piperacillin; SBTPC, Sultamicillin; ICS/LABA, Inhaled corticosteroid/long-acting β-agonists; CPFX, Ciprofloxacin; BMZ, Betamethasone.

^a^ This patient had DIILD during FOLFIRINOX (a combination of 5-fluorouracil, leucovorin, irinotecan, and oxaliplatin) therapy following gemcitabine.

^b^ This patient had DIILD during adriamycin + cyclophosphamide therapy following trastuzumab.

^c^ This patient had DIILD during docetaxel therapy following nivolumab.

^d^ This patient had DIILD during nanoparticle albumin-bound paclitaxel therapy following trastuzumab.

We used 3,554 general Japanese population registered in the integrative Japanese Genome Variation Database (iJGVD) as study controls. Because DIILD is considered a rare adverse event in the population, using a large population control set (healthy individuals) increases the statistical power even while recognizing the (small) potential for misclassification. Population controls have been successfully used in this fashion in previous studies of rare adverse drug reactions and have yielded significant associations [25–28]. In addition, 1,138 patients undergoing chemotherapy but never developed DIILD were recruited as tolerant controls from the NCC biobank in a replication analysis for the two possible SNVs (rs35198919 and rs12625311). This study was approved by the Institutional Review Board of the National Cancer Center (Tokyo, Japan) and the Japanese Foundation for Cancer Research (Tokyo, Japan).

### WGS

WGS was conducted for 26 DNA samples from patients with DIILD. Genomic DNA was extracted from the whole blood or normal tissues, sheared into approximately 350 bp fragments, and used to make a library with the TruSeq Nano DNA Sample Prep Kit (Illumina, San Diego, CA, USA). Sequencing was performed on an Illumina HiSeq X platform using a paired-end 150 bp configuration.

### Mapping, variant calling, filtering, and Sanger sequencing

Adapter sequences were removed by Cutadapt (v1.2.1) [29]. After quality control, reads were mapped to the reference human genome (hg19) using Burrows–Wheeler Aligner (BWA) (ver.0.7.10) [30]. Mapping results were corrected using Picard (ver.1.73) (http://broadinstitute.github.io/picard/) for removing duplicates and Genome Analysis Toolkit (GATK, ver.1.6–13) for local alignment and quality score recalibration. SNV calls were performed with multi-sample calling using GATK [31] and filtered to coordinate with Variant Quality Score Recalibration (passed) and the variant call quality score (≥ 30). Annotations of the variants were based on dbSNP149, CCDS (NCBI, Release 15), RefSeq (UCSC Genome Browser, Feb 2017), Gencode (UCSC Genome Browser, ver. 19), and 1000 Genomes (phase3 release v5). SNVs were further filtered according to the following criteria: (i) A genotype quality score ≥ 20; (ii) Variants in segmental duplication, homopolymer, or repeat regions identified by Repeat Masker or Tandem Repeats Finder were excluded; (iii) SNVs showing significantly different allele frequencies (*P* ≤ 0.05) between 104 Japanese subjects (JPT) in the 1000 Genomes Project and 3,554 Japanese subjects in the iJGVD were excluded. Forty-two candidate SNVs identified by the WGS screening study were verified by Sanger sequencing. In the replication study, genotyping was performed by Sanger sequencing as previously described [32].

### Statistical analysis

In the screening (WGS) and replication study, a case (patients with DIILD)-control (3,554 general Japanese individuals in iJGVD) [33] association study was performed using the Fisher’s exact test in an allele frequency model. The significance levels after Bonferroni correction for multiple testing were *P* = 7.60 × 10^−8^ (0.05/658,234) in the screening study and *P* = 1.47 × 10^−3^ (0.05/34) in the replication study because eight SNVs were highly linked (*r*^*2*^ = 1) in the 42 candidate SNVs. The odds ratios (ORs) and confidence intervals (CIs) were calculated using the non-risk allele as a reference. All statistical analyses were carried out using R statistical environment version 3.3.1 (http://www.r-project.org/) or the BellCurve for Excel (Social Survey Research Information Co., Ltd., Tokyo, Japan).

## Results

### Clinical characteristics of patients with drug-induced ILD

The clinical characteristics of the 44 patients who developed DIILD are summarized in Table 1. The median age was 62 years (range: 36–80 years) and 25 (57%) were female. Seven (16%) and 21 (48%) patients had a history of ILD and smoking, respectively. We enrolled patients with lung cancer (23 patients), breast cancer (7 patients), pancreatic cancer (6 patients), melanoma (5 patients), and gastric cancer (3 patients). The causative drugs of ILD in the 44 patients were found to be nivolumab (14 patients), EGFR-TKI (gefitinib or erlotinib; 13 patients), trastuzumab plus paclitaxel (7 patients), gemcitabine (3 patients), gemcitabine plus nanoparticle albumin-bound paclitaxel (2 patients), FOLFIRINOX (a combination of 5-fluorouracil, leucovorin, irinotecan, and oxaliplatin; 1 patient), adriamycin plus cyclophosphamide (1 patient), trastuzumab emtasine (1 patient), docetaxel (1 patient), and nanoparticle albumin-bound paclitaxel (1 patient). In all patients with DIILD, the causative drugs were withdrawn after the diagnosis of ILD, and most received treatment for ILD as shown in Table 1.

### WGS and identification of candidate genetic biomarkers for the risk of DIILD

To identify genetic susceptibility loci for DIILD, we performed WGS using the germline DNA of 26 patients who developed DIILD during anticancer treatment (Table 1). The mean number of SNVs per patient was 4,042,041 with a 35× average depth. Variants were filtered using an in-house program as described in the Materials and methods; a total of 658,234 SNVs were analyzed in the 26 patients. We next conducted a case-control association study between the 26 cases and the 3,554 (maximum) general Japanese population controls registered in the iJGVD to identify SNVs associated with DIILD as a screening study. Although no SNVs reached a significance level of *P* < 7.60 × 10^−8^ (see Materials and methods), we observed 42 SNVs showing *P* < 0.0001 (3.31 × 10^−6^ < *P* < 9.94 × 10^−5^) as shown in S1 Table.

### Replication and combined study using additional patients

**Fig 1:**
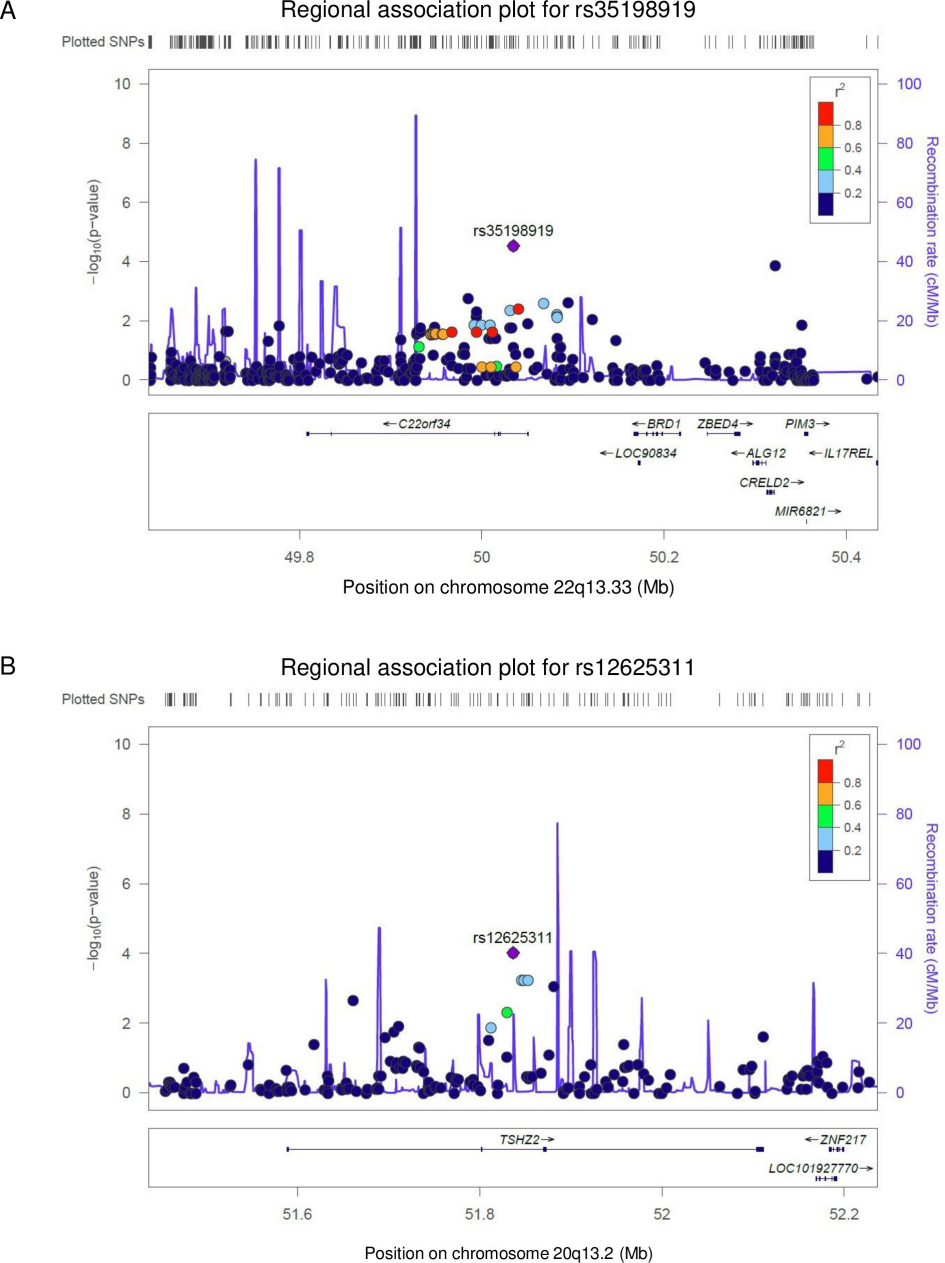
Regional association plots of two SNVs associated with DIILD. Upper panel; *P* values of the SNVs are plotted (as–log_10_*P* values) against their physical location on chromosome 22 (A) and 20 (B). The genetic recombination rates estimated from the 1,000 Genomes samples (Japanese in Tokyo, JPT + Chinese in Beijing, CHB) are shown with a blue line. SNV color indicates linkage disequilibrium (LD) with rs35198919 (A) and rs12625311 (B) according to a scale from *r*^*2*^ = 0 to 1 based on pairwise *r*^*2*^ values from the Asian (ASN) data from the 1,000 Genomes Project. Lower panel; gene annotations from the University of California Santa Cruz genome browser.

The associations of 42 SNVs showing *P* < 0.0001 in the screening study were further investigated using an independent cohort consisted of 18 DIILD cases (Table 1, S1 Table). We performed genotyping of the 42 SNVs by the Sanger sequencing method using the above 18 cases; however, we could not observe the SNV that reached the significance level of *P* = 1.47 × 10^−3^ (see Materials and methods) as shown in S1 Table. However, in the combined analysis of the screening and replication studies for the 42 SNVs, we found that the associations of two SNVs, rs35198919 in intron 1 of *C22orf34* and rs12625311 in intron 1 of *TSHZ2*, with DIILD were stronger than those in the screening study (*P*_*combined*_ = 1.87 × 10^−5^, odds ratio (OR) = 4.15, 95% CI; 2.36–7.31, and *P*_*combined*_ = 5.16 × 10^−5^, OR = 2.43, 95% CI; 1.59–3.71, respectively, Table 2, Fig 1).

**Table 2:** Association results of two SNVs with smaller *P* values in the combined analysis of screening and replication studies than those in the screening study.

Chr.	SNP ID	Position^a^	Gene	Allele (1/2)^b^	Location	Stage	Risk allele	Case					Control^c^					*P* value	Odds ratio [95% CI]
								Allele		Allele Frequency		RAF	Allele		Allele Frequency		RAF		
								1	2	1	2		1	2	1	2			
22	rs35198919	50,034,703	*C22orf34*	T/C	Intron	Screening	C	41	11	0.79	0.21	0.21	6,725	333	0.95	0.05	0.05	2.93 x 10^−5^	5.42
																			[2.76–10.64]
						Replication		32	4	0.89	0.11	0.11						8.94 x 10^−2^	2.52
																			[0.89–7.18]
						Combined		73	15	0.83	0.17	0.17						1.87 x 10^−5^	4.15
																			[2.36–7.31]
20	rs12625311	51,836,793	*TSHZ2*	T/C	Intron	Screening	C	21	31	0.40	0.60	0.60	4,755	2,349	0.67	0.33	0.33	9.59 x 10^−5^	2.99
																			[1.71–5.21]
						Replication		19	17	0.53	0.47	0.47						7.75 x 10^−2^	1.81
																			[0.94–3.49]
						Combined		40	48	0.45	0.55	0.55						5.16 x 10^−5^	2.43
																			[1.59–3.71]

Chr., chromosome; RAF, risk allele frequency; CI, confidence interval.

^a^ Based on GRCh37 genome assembly.

^b^ Reference allele (GRCh37) was defined as allele 1.

^c^ The same controls were used in screening, replication, and combined analysis.

### Subgroup analysis for the identification of genetic markers for EGFR-TKI-induced ILD

To identify the genetic marker(s) for EGFR-TKI-induced ILD, we next performed an association study using the 13 patients with EGFR-TKI-induced ILD (cases) and the abovementioned controls from the iJGVD as a subgroup analysis. We observed that seven SNVs were possibly associated with EGFR-TKI-induced ILD, with *P* < 0.00001 as shown in Table 3, however, they showed no or weak associations in the 13 patients with nonEGFR-TKI-induced ILD (*P* = 0.25–1).

**Table 3:** Association results of seven SNVs with *P* < 0.00001 in the EGFR-TKI-induced ILD subgroup analysis.

Chr.	SNP ID	Position^a^	Gene	Allele (1/2)^b^	Location	Risk allele	EGFR-TKI-induced ILD												nonEGFR-TKI-induced ILD	
							Case					Control					*P* value	Odds ratio [95% CI]	*P* value	Odds ratio [95%CI]
							Allele		Allele Frequency		RAF	Allele		Allele Frequency		RAF				
							1	2	1	2		1	2	1	2					
5	rs75399069	24,664,646	Intergenic	A/C		C	19	7	0.73	0.27	0.27	6,921	171	0.98	0.02	0.02	2.39 x 10^−6^	14.91	4.71 x 10^−1^	1.62
																		[6.19–35.94]		[0.22–12.02]
5	rs417168	135,216,860	*SLC25A48*	T/C	Intron	C	23	3	0.88	0.12	0.12	7,086	6	0.999	0.001	0.001	3.58 x 10^−6^	154.04	1.00	0
																		[36.31–653.49]		
5	rs442281	135,216,861	*SLC25A48*	G/A	Intron	A	23	3	0.88	0.12	0.12	7,086	6	0.999	0.001	0.001	3.58 x 10^−6^	154.04	1.00	0
																		[36.31–653.49]		
20	rs17690253	16,894,293	Intergenic	T/G		G	19	7	0.73	0.27	0.27	6,892	200	0.97	0.03	0.03	6.53 x 10^−6^	12.70	1.00	0
																		[5.28–30.54]		
6	rs184448987	103,169,971	Intergenic	C/A		A	21	5	0.81	0.19	0.19	7,023	73	0.99	0.01	0.01	7.61 x 10^−6^	22.91	1.00	0
																		[8.41–62.40]		
2	rs10165147	154,697,039	Intergenic	C/G		G	9	17	0.35	0.65	0.65	5,412	1,686	0.76	0.24	0.24	8.22 x 10^−6^	6.06	2.45 x 10^−1^	1.70
																		[2.70–13.63]		[0.76–3.82]
2	rs1348851	178,418,677	Intergenic	A/G		A	6	20	0.23	0.77	0.23	134	6,968	0.02	0.98	0.02	8.59 x 10^−6^	15.60	9.99 x 10^−1^	0
																		[6.17–39.47]		

Chr., chromosome; RAF, risk allele frequency; CI, confidence interval.

^a^ Based on GRCh37 genome assembly.

^b^ Reference allele (GRCh37) was defined as allele 1.

## Discussion

The development of predictive marker(s) for the risk of life-threatening toxicities related to cancer treatment, including ILD, based on germline mutation or variation is important in order to provide safe treatment regimens for patients with cancer. This study is the first WGS that attempts to identify common or rare genetic variants associated with DIILD in the Japanese population. The SNV strongly associated with DIILD was not identified in this study; however, we identified two independent loci showing possible associations with DIILD (Table 2, Fig 1). Moreover, subgroup analysis using patients with EGFR-TKI-induced ILD suggested a possible association of seven SNVs with EGFR-TKI-induced ILD (Table 3). To enlarge the sample size, we further added patients undergoing chemotherapy after cancer diagnosis but never developed DIILD as a control for a replication cohort and analyzed for rs35198919 and rs12625311 (S2 Table). The association results of the replication and combined studies were comparable with those obtained from the original control set (general Japanese population) as shown in Table 2 and S2 Table.

The most strongly associated SNV from the combined results of the screening and replication studies was rs35198919 (*P*_combined_ = 1.87 × 10^−5^, OR = 4.15), located in *C22orf34* on chromosome 22q13.33. Although the function of *C22orf34* remains to be clarified, the gene expression of *C22orf34* in human lung is higher compared with other human tissues according to the Genotype-Tissue Expression (GTEx) Analysis Release V7 [34]. To effectively clarify the mechanism of DIILD, we performed expression quantitative trait locus (cis-eQTL) analysis for rs35198919 and observed a cis-regulatory effect on *C22orf34* expression in whole blood and thyroid (*P* = 5.80 × 10^−6^ and 6.50 × 10^−6^, respectively, S1 Fig). However, further studies are required to clarify the functional associations of *C22orf34* with DIILD.

rs12625311 demonstrated a stronger association in the combined study and is located in intron 1 of *TSHZ2*, which encodes a transcriptional repressor that suppresses the transcriptional activity of genes involved in tumor development and growth [35, 36]. Single nucleotide polymorphisms (SNPs) in *TSHZ2* are known to be involved in Stevens-Johnson syndrome/toxic epidermal necrolysis with severe ocular complications (SJS/TEN with SOCs) in the Japanese population [37]. ILD has been reported to be one of the complications in patients with SJS [38, 39], suggesting a common mechanism between SJS and DIILD involving a pathway regulated by TSHZ2. However, further validation studies and functional analyses are needed to evaluate the true association. DIILD, including EGFR-TKI-induced ILD, is more common in the Japanese population [40, 41]. The frequency of the risk allele (C) in rs12625311 is higher in the Japanese population (36%) compared with other ethnic populations (South Asian: 19%, African: 18%, American: 3% and European: 2% in the 1000 Genomes Project), suggesting that the difference in the above allele frequency may cause interethnic differences in the frequency of DIILD.

We next examined the association of the two SNVs (rs35198919 and rs12625311) in patients with a history of ILD and patients that had never had ILD before. The above two SNVs showed larger odds ratios in patients with a history of ILD (rs35198919: OR = 5.51, rs12625311; OR = 7.42) compared with those that had never had ILD (rs35198919: OR = 3.91, rs12625311; OR = 2.02). These findings suggested that these two SNVs may be strong predictors for DIILD in patients with a history of ILD (S3 Table). We further carried out a subgroup analysis of the tumor type for the two possible SNVs (rs35198919 and rs12625311). Although none of the tumor types showed smaller *P* values compared with those in the original analysis, lung and pancreatic cancers showed larger ORs in rs35198919 and rs12625311, and gastric cancer also showed larger ORs in rs12625311 (S4 Table), suggesting that these SNVs may be specific predictors for the risk of DIILD in patients with lung, pancreatic, and gastric cancers.

In the subgroup analysis of EGFR-TKI-induced ILD, we identified seven candidate SNVs that could be predictive markers for the risk of EGFR-TKI-induced ILD (Table 3). Although none of these SNVs showed a significant association with nonEGFR-TKI-induced ILD, four (rs417168, rs442281, rs75399069, and rs10165147) were included in the 42 candidate SNVs identified in the screening study using the 26 patients with DIILD (S1 Table). Moreover, the associations of these four SNVs in the 13 patients with EGFR-TKI-induced ILD were stronger compared with those of the 26 patients with ILD induced by either EGFR-TKI or nonEGFR-TKI. Hence, these four SNVs may be candidate predictive markers for the risk of EGFR-TKI-induced ILD rather than common predictive markers for the risk of DIILD. Of these four SNVs, rs10165147 is located 31 kb upstream of the polypeptide N-acetylgalactosaminyltransferase 13 (*GALNT13*) (S2 Fig). GALNT13 is a member of the UDP-N-acetyl-alpha-D-galactosamine:polypeptide N-acetylgalactosaminyltransferase family, which initiate the O-linked glycosylation of mucins [42]. Mucin 5B, oligomeric mucus/gel-forming (*MUC5B*) has been reported to be one of the candidate genetic markers for ILD [43, 44]. Although further analysis is required to clarify the functional importance of *GALNT13* in DIILD, the difference in efficiency of the O-linked glycosylation of mucins by *GALNT13* may cause interindividual differences in the susceptibility to DIILD. Furthermore, we investigated the association between *MUC5B* and DIILD and found that rs565375327 showed the lowest *P* value [screening cohort (26 patients); *P* = 3.58 × 10^−3^, EGFR-TKI-induced ILD subgroup (13 patients); *P* = 4.74 × 10^−4^] of the SNVs in *MUC5B*, suggesting that *MUC5B* may be a promising predictive marker for the risk of DIILD, particularly for EGFR-TKI-induced ILD as well as *GALNT13*.

Surfactant protein A1 (*SFTPA1*) and surfactant protein A2 (*SFTPA2*) have been suggested as susceptibility genes for IPF [45, 46]. In the current study, SNVs in these candidate genes showed no or weak associations with DIILD (*P* = 2.29 × 10^−1^–9.99 × 10^−1^). It is reported that gefitinib inhibits cyclin G–associated kinase (GAK), as well as EGFR signaling [47], and kinase deficiency of GAK causes the neonatal death of mice due to respiratory dysfunction [48]. However, no SNVs in *GAK* showed a significant association with EGFR-TKI-induced ILD (EGFR-TKI-induced ILD subgroup; *P* = 5.76 × 10^−2^–9.99 × 10^−1^) in our study. In addition, although the expression level of Mucin 4, cell surface associated (*MUC4*) has been reported to be increased in IPF[49], we observed no association between *MUC4* and EGFR-TKI-induced ILD (*P* = 1.74 × 10^−1^–9.99 × 10^−1^). Further validation analysis using a larger number of patients is required to clarify the effects of these variants on the risk of DIILD.

Previous candidate gene approaches have reported the positive association of HLA alleles with DIILD [23, 24]. However, these results are unlikely to be fully replicated, and some of the studies failed to confirm the positive association of DIILD with these HLA alleles. Further validation studies using independent cohorts are needed to confirm the true association of the HLA alleles with DIILD. On the other hand, in our study, we carried out a WGS approach to identify novel candidate variants that might be involved in the unknown mechanism underlying DIILD. Because the sample size used in our study was not large enough, we could not identify the variants that reached the significance level after Bonferroni correction. Similar to the above candidate genes, further validation studies using larger sample size cohorts are needed to confirm the true association of the variants identified in this study with DIILD. To establish a clinically useful prediction system for the risk of DIILD, the integration of the results from the candidate gene and WGS approaches is important. Furthermore, combination analysis of variants identified using the WGS approach with previously identified variants using candidate gene approach, including HLA alleles, may prove to have cumulative effects on the risk of DIILD.

In conclusion, using a WGS approach, we identified two SNVs as possible genetic susceptibility factors for the risk of DIILD. Although the mechanism underlying DIILD should be further investigated by using a larger number of samples and molecular analysis, our results provided a possibility of prediction of the risk of DIILD that could lead to better prognosis and quality of life for patients with cancer.

## Supporting information

S1 Fig Results of eQTL analysis for rs35198919. Expression quantitative trait locus (eQTL) analysis using GTEx Portal (https://gtexportal.org/home/) indicated that the C allele (risk allele) of rs35198919 was significantly associated with decreased expression of *C22orf34* in whole blood (*P* = 5.80 × 10^−6^) (A) and thyroid (*P* = 6.50 × 10^−6^) (B).
(TIF)

S2 Fig Regional association plot for rs10165147. Upper panel; *P* values of SNVs are plotted (as–log_10_*P* values) against their physical location on chromosome 2q23.3-q24.1. The genetic recombination rates estimated from 1,000 Genomes samples (Japanese in Tokyo, JPT + Chinese in Beijing, CHB) are shown with a blue line. SNV color indicates the linkage disequilibrium (LD) with rs10165147 according to a scale from *r*^*2*^ = 0 to 1 based on pairwise *r*^*2*^ values from the Asian (ASN) data in the 1,000 Genomes Project. Lower panel; gene annotations from the University of California Santa Cruz genome browser.
(TIF)

S1 Table Association results of screening, replication, and combined studies for the 42 SNVs with *P* < 0.0001 in the screening study. (XLSX)

S2 Table Replication analysis of rs35198919 and rs12625311 using tolerant controls. (XLSX)

S3 Table Subgroup analysis of rs35198919 and rs12625311 according to the history of ILD. (XLSX)

S4 Table Subgroup analysis of rs35198919 and rs12625311 by cancer type. (XLSX)
