# Supplementary figures and images for: Whole genome sequencing to identify predictive markers for the risk of drug-induced interstitial lung disease
Source: PLoS One. 2019 Oct 4;14(10):e0223371. doi: 10.1371/journal.pone.0223371 (PMC6777826; doi:10.1371/journal.pone.0223371)

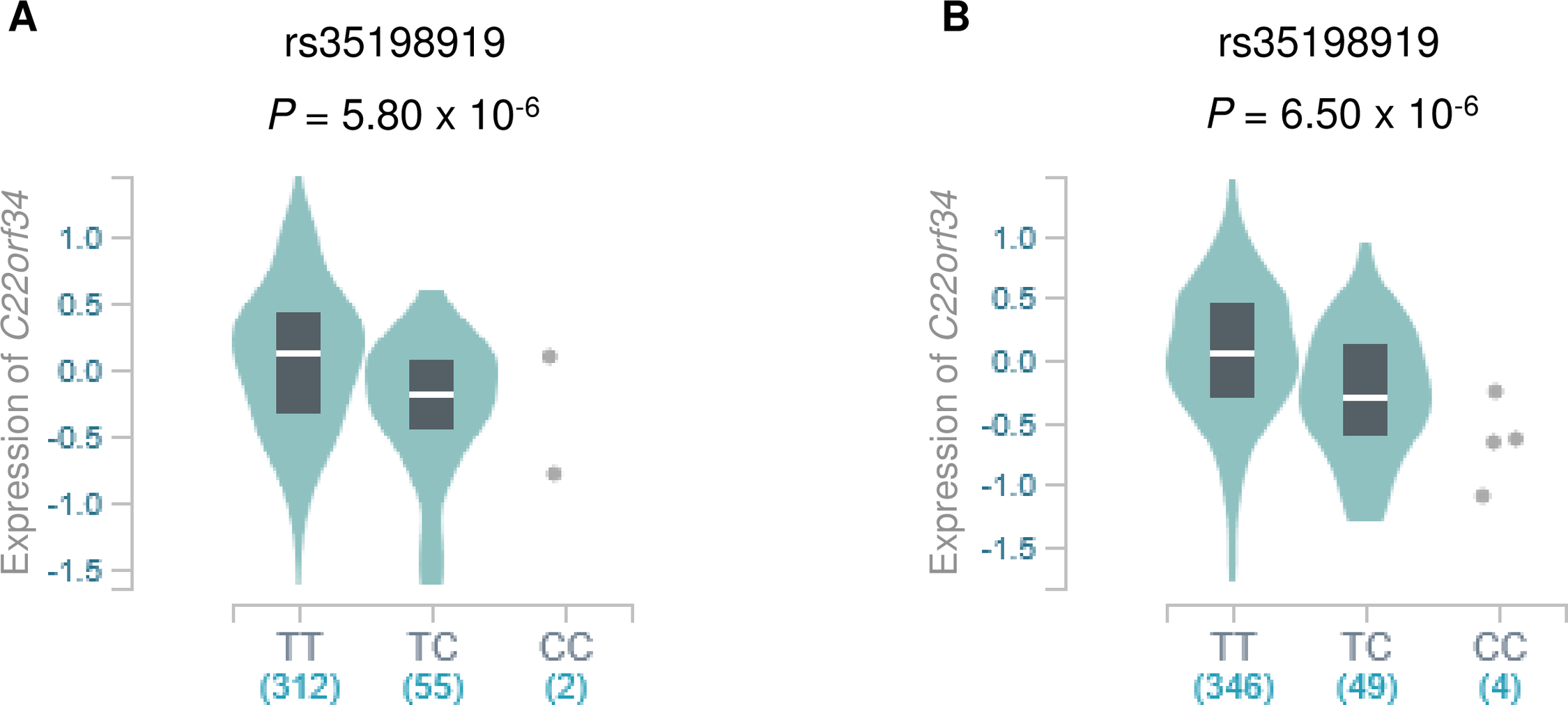

Supplement: S1 Fig — Expression quantitative trait locus (eQTL) analysis using GTEx Portal (https://gtexportal.org/home/) indicated that the C allele (risk allele) of rs35198919 was significantly associated with decreased expression of C22orf34 in whole blood (P = 5.80 × 10^−6^) (A) and thyroid (P = 6.50 × 10^−6^) (B). (TIF) [file pone.0223371.s001.tif]

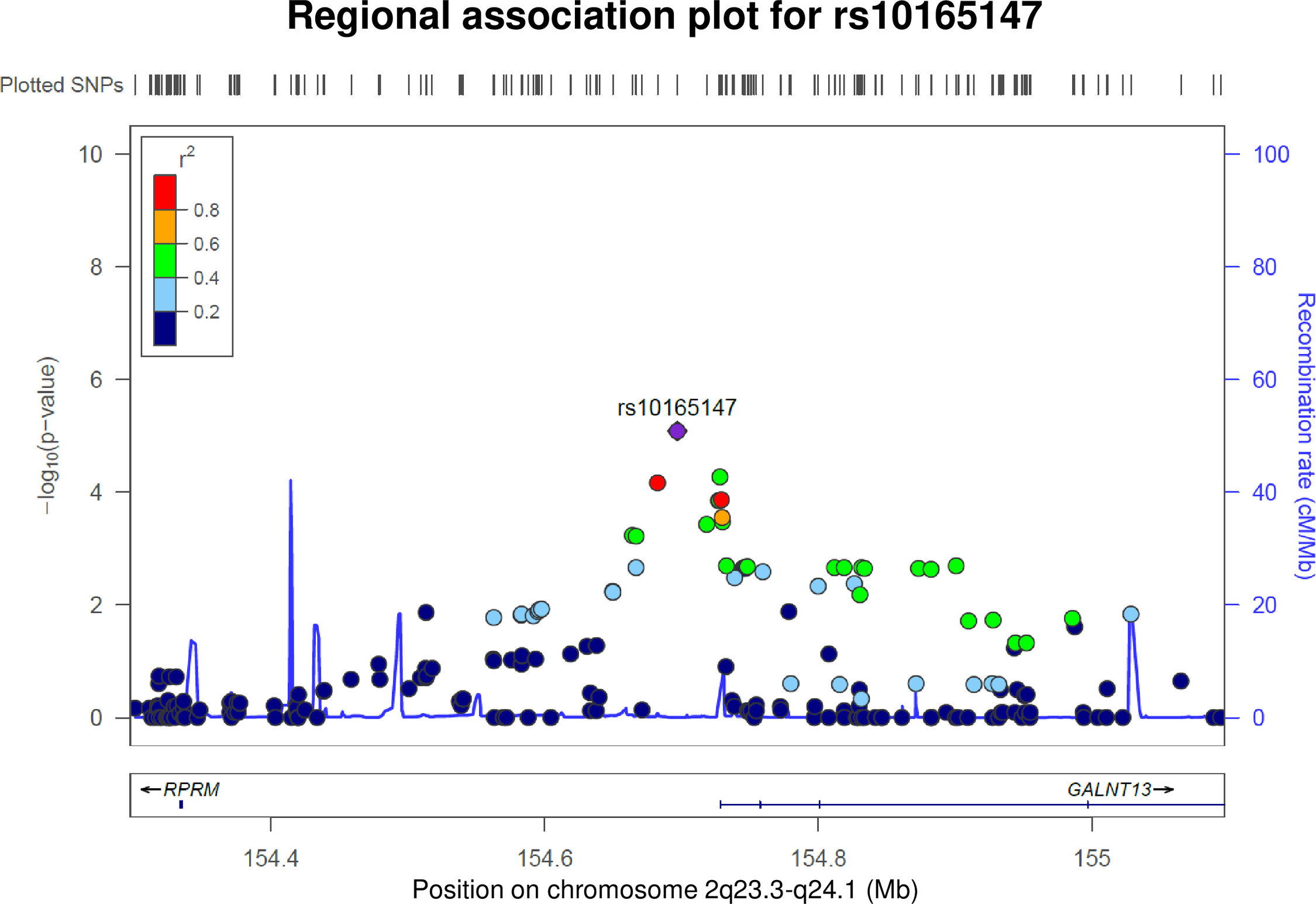

Supplement: S2 Fig — Upper panel; P values of SNVs are plotted (as–log10P values) against their physical location on chromosome 2q23.3-q24.1. The genetic recombination rates estimated from 1,000 Genomes samples (Japanese in Tokyo, JPT + Chinese in Beijing, CHB) are shown with a blue line. SNV color indicates the linkage disequilibrium (LD) with rs10165147 according to a scale from r2 = 0 to 1 based on pairwise r2 values from the Asian (ASN) data in the 1,000 Genomes Project. Lower panel; gene annotations from the University of California Santa Cruz genome browser. (TIF) [file pone.0223371.s002.tif]
